# Socioeconomic Disparities in Diagnosis-to-Treatment Time Among Patients Diagnosed With Breast Cancer in Saudi Arabia: A Cross-Sectional Study in a Tertiary Care Center

**DOI:** 10.7759/cureus.70533

**Published:** 2024-09-30

**Authors:** Ghader Jamjoum, Sarah Y Bahowarth, Hussain A Alkhalifah, Nawaf H Alshehri, Osman M Melibari, Wed H Youssuf, Amal A Alshehri, Elham M Metwally

**Affiliations:** 1 General Surgery, Hepato-Pancreato-Biliary (HPB) Surgery, Surgical Oncology, King Abdulaziz University Hospital, Jeddah, SAU; 2 Medicine, King Abdulaziz University Hospital, Jeddah, SAU; 3 Medicine, King Abdulaziz University Faculty of Medicine, Jeddah, SAU

**Keywords:** breast cancer, cancer, disparities, oncology, socioeconomic

## Abstract

Introduction

Despite Saudi Arabia’s' free healthcare system, breast cancer (BC) has a major impact on affected individuals. Previous studies have shown that socioeconomic variables could contribute to inequities in receiving treatment. Although early detection and treatment are essential, delays are frequently influenced by either insurance status or other socioeconomic variables. Assessing characteristics that influence the duration of BC treatment for Saudi women will aid in improving health equity and lowering system costs.

Methods

This was a cross-sectional study that included all female patients who were diagnosed with BC between 2016 and 2023 at a tertiary care center. All patients were contacted by phone calls to fill out a questionnaire.

Results

A total of 113 females were included; the mean age at the time of diagnosis with BC was 48.88±10.97 years, and the majority were Saudis (58.4%). Additionally, the median duration for treatment initiation was 28 (15.50-45.50) days from the date of diagnosis. Factors influencing the time for initiating the treatment included nationality, as non-Saudis took longer to receive their treatment (27.00 (13.00-39.25) days vs. 30.00 (18.00-59.00) days, p = 0.176). Moreover, patients living further from the hospital demonstrated a delay in receiving treatment compared to those living near the hospital. However, the relation was not statistically significant.

Conclusion

Our study investigated the demographic disparities among BC patients. Our results showed that some variables contributed to a delay in treatment initiation, including nationality and distance from the hospital, which suggest further areas for investigation. We recommend further studies be conducted with a larger sample size to improve accessibility and reduce treatment delays for BC patients.

## Introduction

One of the major health challenges facing the world today is breast cancer (BC). Breast cancer caused almost 685,000 deaths globally in 2020 [1, 2]. This shows how much women's health is impacted by BC worldwide, and the Kingdom of Saudi Arabia (KSA) is no exception [3]. As is shown in the past two decades, the BC incidence rate has increased by almost 10-fold in KSA, and the incidence rate is projected to increase due to population growth and aging [4, 5]. The fact that BC is still the most significant cause of mortality for Saudi women must be emphasized [6, 7]. Despite early detection, screening, and systemic therapy improvements, variations in BC outcomes can continue to exist among different population groups, resulting from a complex interplay of biological and social factors in the health system due to various socioeconomic positions [8, 9]. Social determinants of health are a topic of discussion in national debates about health disparities and health equity. Socioeconomic status, education, physical environment, occupation, social support networks, and access to healthcare are all examples of social determinants of health. Addressing socioeconomic determinants of health is crucial to improve health and lessen persistent inequities in health and healthcare [10, 11]. An essential tactic for improving the prognosis of the disease is the early detection and timely treatment of BC [12].

It has been studied how various socioeconomic factors, specifically the impact of insurance, affected treatment delays. Without insurance, the lack of screening, access to care, and delays in diagnosis result in later stages of the disease upon diagnosis and, thus, worse survival rates [2, 13, 14]. According to previous research, patients with the longest treatment times tended to be younger, poorer, uninsured, less educated, and in a more advanced stage of the disease at the time of diagnosis [15].

Currently, few studies have been done in KSA and the Middle East regarding disparities in BC from the time of diagnosis to treatment and what interventions can effectively reduce these disparities. This analysis could lead to policy recommendations that improve health equity and, consequently, reduce health system costs in KSA. Moreover, there will be a more manageable way to distribute clinical knowledge and health infrastructure across various user populations. We believe it will significantly contribute to current local and regional data by analyzing and quantifying biological, social, and health factors in Saudi women. Therefore, this study aims to assess the socioeconomic disparities in BC patients and their effect on the timing of receiving treatment in women diagnosed with BC in Jeddah, Saudi Arabia.

## Materials and methods

Study design, sitting, and population

This was a cross-sectional study that included all female patients diagnosed with BC between 2016 and 2023 at a tertiary care center. Patients who decided to receive the diagnosis from outside our center and those who had metastasis to any organ were excluded.

Of 386 patients diagnosed with BC during the study period, all were contacted by phone to complete a questionnaire. Each patient was contacted up to three times on three different days and times to ensure the highest response rate possible. Deceased patients who had metastasis or chose not to participate were excluded from the study.

Data collection

All cancer-related patient data, including date of diagnosis, histopathology of cancer, tumor, node, metastasis (TNM) staging, hormonal receptors for the tumor, date of receiving treatment, and type of initial treatment received, were obtained retrospectively through the hospitals' database. After that, patients were contacted via phone calls to complete a questionnaire that inquired about demographic and social data.

Questionnaire

The study’s questionnaire was adopted from pre-validated questionnaires after reviewing several studies and was constructed based on the current study’s objectives [16, 17]. The questionnaire inquired about data regarding nationality, marital status, number of children, educational level, employment status, type and location of residence, household incomes, whether they had insurance if they needed it to take a loan after receiving the diagnosis, family history of cancer, smoking, and whether they had any comorbidities (Appendix A).

To ensure the suitability of the questionnaire items, three general surgery consultants performed face validation by reviewing the questionnaire to ensure its accuracy in measuring the study objectives; modifications were made accordingly to enhance the questionnaire.

Data analysis

Microsoft Excel 2021 (Microsoft Corp., Redmond, WA) was used for data entry, and IBM SPSS Statistics, version 27 (IBM Corp., Armonk, NY) was used for data analysis and coding. Categorical data, including demographic and personal characteristics, cancer details, and management details, are expressed as frequencies. Normally distributed continuous data such as age at diagnosis were expressed as mean and standard deviation (SD). In contrast, medians and interquartile ranges (IQR) were used for data that were not normally disputed, such as the number of days between diagnosis and receiving the treatment. Bivariate analysis was conducted using the Mann-Whitney U test and the Kruskal-Wallis test to identify the demographic and personal factors associated with a longer duration to receive the treatment. Statistical significance was set at p<0.05.

Ethical approval

This research was authorized by the institutional review board of King Abdulaziz University Hospital, Jeddah, Saudi Arabia (reference number 298-23), and complied with the Declaration of Helsinki's requirements. Every participant gave verbal informed consent prior to filling out the questionnaire and was told of the study objectives.

## Results

Sociodemographic and clinical characteristics of the study group

Out of the 386 patients contacted, 160 did not answer, and 27 refused to participate. After applying the exclusion criteria, 49 patients were excluded. In the end, 113 patients were included in the current study.

The mean age at diagnosis with BC was 48.88±10.97 years, and most of them were Saudis (n = 66, 58.4%). Most were married (n = 84, 74.3%) and had children (n = 79, 69.9%). Only 30 patients (26.5%) were employed at the time of diagnosis. However, after receiving the diagnosis, 10 (33.33%) changed their employment status. Three resigned from their job, three got their contract terminated by their employer, two retired, and two changed their position at work to a more comfortable one. Additionally, out of the 22 patients who continued to work, most (n = 16, 72.7%) had to take some days off, with the majority taking more than two weeks off (n = 13, 81.3%). Ninety-one patients lived inside the city (80.5%), while 22 lived outside (19.5%). Most lived in a rental home or apartment (n = 61, 54.0%), and most lived more than 20 minutes away from the hospital (n = 58, 51.3%). Furthermore, most of the sample had a low household income, as less than 3,000 Saudi Riyals (n = 27, 23.9%) and 3,001-5,000 Saudi Riyals (n = 26, 23.0%) were the most reported household incomes. Only 13 patients (11.5%) reported having a personal car and can drive themselves to the hospital. At the same time, the rest depended on someone else to drive them, as 65 patients (57.5%) had a relative or a friend drive them, 20 (17.7%) took public transportation, and 15 (13.3%) had a personal driver. Moreover, most did not have medical insurance (n = 92, 81.4%), and 31 patients (27.4%) reported having to take a loan after their diagnosis. Table 1 demonstrates the sociodemographic characteristics and personal data of all the patients.

**Table 1:** Sociodemographic characteristics and personal data of the participants The numerical data are expressed as mean ± standard deviation, and others are expressed as numbers (N) & percentages (%). SD: standard deviation; PhD: Doctor of Philosophy

Variables	Mean	SD
Age at the time of diagnosis (years)	48.88	10.97
	N	%
Marital status		
Married	84	74.3%
Single	8	7.1%
Divorced	8	7.1%
Widow	13	11.5%
Nationality		
Saudi	66	58.4%
Non-Saudi	47	41.6%
Do you have children?		
Yes	79	69.9%
No	34	30.1%
Number of children		
1-2	18	23.7%
3-5	47	61.8%
More than 5	11	14.5%
Were you a smoker at the time of diagnosis		
Yes	6	5.3%
No	107	94.7%
Educational level		
Did not receive an education	17	15.0%
Primary school	12	10.6%
Middle school	11	9.7%
High school	20	17.7%
Diploma	8	7.1%
Bachelors	36	31.9%
Masters or PhD	9	8.0%
Were you employed at the time of diagnosis?		
Yes	30	26.5%
No	83	73.5%
Did your employment status change after diagnosis?		
No change	20	66.7%
I retired	2	6.7%
I resigned	3	10.0%
My contract was terminated	3	10.0%
I changed my position at work	2	6.7%
Did you take any days off?		
Yes	16	72.7%
No	6	27.3
How many days did you take off?		
Two weeks or less	3	18.8%
More than two weeks	13	81.3%
How helped and supported you during those times		
Husband	44	38.9%
Parents	5	4.4%
Children	43	38.1%
Siblings	16	14.2%
Relatives	2	1.8%
I did not have any support	3	2.7%
Residence		
Owned	40	35.4%
Rental	61	54.0%
I live with someone else	12	10.6%
Where do you live?		
Jeddah	91	80.5%
Outside Jeddah	22	19.5%
How far is the hospital from your residence?		
Less 20 min	55	48.7%
20 min – 60 min	46	40.7%
More than 60 min	12	10.6%
How do you visit the hospital?		
I drive myself	13	11.5%
I have a personal driver	15	13.3%
A relative or a friend drives me	65	57.5%
I take public transportation	20	17.7%
Household monthly income (Riyal)		
Less than 3,000	27	23.9%
3,000-5,000	26	23.0%
5,001-10,000	24	21.2%
10,001-15,000	16	14.2%
15,001-20,000	8	7.1%
More than 20,000	12	10.6%
Do you have a family history of cancer?		
Yes	46	40.7%
No	67	59.3%
Do you have insurance?		
Yes	21	18.6%
No	92	81.4%
Did you need to take out a loan?		
Yes	31	27.4%
No	82	72.6%
Comorbidities		
Yes	36	31.9%
No	77	68.1%

Details regarding cancer diagnosis and treatment received

Out of the total sample, the most reported pathology for cancer was invasive ductal carcinoma (IDC) (n = 99, 87.2%), followed by invasive lobular carcinoma (ILC) (n = 12, 10.8%). Using the TNM classification, most tumors were T2 (n = 68, 60.2%) and N0 (n = 57, 50.0%). Moreover, after the biopsy, most tumors were ER and PR positive (n = 86, 76.1%) and HER2 negative (n = 69, 61.1%). The median number of days for patients to get their treatment from the day of diagnosis was 28 (15.50-45.50) days, and the initial treatments included surgery (n = 55, 48.7%), neoadjuvant chemotherapy (n = 55, 48.7%), and hormonal therapy (n = 3, 2.7%). Table 2 demonstrates the details of the cancer and the initial treatment received.

**Table 2:** Cancer and treatment details The numerical data are expressed as median and interquartile ranges, and others are expressed as numbers (N) & percentages (%).

Variables	Median	Interquartile range (IQR)
The period from diagnosis to receiving treatment (days)	28	15.50-45.50
	N	%
Grade		
Grade 1	17	16.5%
Grade 2	69	67.0%
Grade 3	17	16.5%
Pathology		
Invasive ductal carcinoma (IDC)	99	87.2%
Invasive lobular carcinoma (ILC)	12	10.8%
Estrogen receptor (ER)		
Positive	86	76.1%
Negative	27	23.9%
Progesterone receptor (PR)		
Positive	86	76.1%
Negative	27	23.9%
Human epidermal growth factor receptor 2 (HER2)		
Positive	42	37.2%
Negative	69	61.1%
Equivocal	2	1.8%
Tumor (T) stage		
T1	27	23.9%
T2	68	60.2%
T3	16	14.2%
T4	1	0.9%
Node (N) stage		
N0	57	50.9%
N1	37	33.0%
N2	11	9.8%
N3	7	6.3%
Cancer stage		
Stage 1	12	10.7%
Stage 2	72	64.3%
Stage 3	28	25.0%
First treatment received		
Surgery	55	48.7%
Chemotherapy	55	48.7%
Endocrine therapy	3	2.7%

Demographic and personal factors affecting the time for receiving treatment

The median duration in days for receiving the treatment was lower among Saudis compared to non-Saudis (27.00 (13.00-39.25) days vs. 30.00 days (18.00-59.00), p=0.176). Additionally, single women showed the most extended duration, as they took a median of 55.00 (11.50-183.25) days when compared to widows, which took 29 (13.00-78.50) days, married women, which took 28 (16.00-39.75) days, and divorced women, which took 24.50 (14.75-35.50) days (p = 0.616). Those who had children were shown to have a shorter duration compared to those who didn’t (26.00 (15.00-39.00) days vs. 34.00 (16.75-63.25) days, p = 0.101). However, there was no significant difference when assessing the effect of the number of children. Moreover, the duration was almost similar between patients who lived outside the city and those who lived inside (26.00 (17.75-34.00) days vs. 30.00 (14.00-47.00) days, p = 0.550). However, patients who lived more than 60 minutes away from the hospital showed the longest duration compared to those living 20-60 minutes and less than 20 minutes away (32.00 (14.25-86.50) days vs. 25.00 (14.75-36.75) days vs. 31.00 (17.00-46.00) days, p = 0.343). Although the household income did not affect the duration of the treatment, patients who did not have medical insurance were shown to have a longer duration than those with insurance (29.50 (17.00-46.00) days vs. 22.00 (10.00-37.50) days, p = 0.234). Table 3 demonstrates the relationships between the different demographic and personal data with the mean duration to receive the initial treatment for BC.

**Table 3:** Relationship between the study variables and the duration between diagnosis and treatment in days The numerical data are expressed as medians and interquartile ranges, and others are expressed as numbers (N) and percentages (%). The p-value was significant at <0.05. N: number; %: percentage; IQR: interquartile range; PhD: Doctor of Philosophy

	N	%	Number of days between diagnosis and receiving treatment in days (Median (IQR))	p-value
Marital status				
Married	84	74.3%	28 (16.00-39.75)	0.616
Single	8	7.1%	55.00 (11.50-183.25)	
Divorced	8	7.1%	24.50 (14.75-35.50)	
Widow	13	11.5%	29 (13.00-78.50)	
Nationality				
Saudi	66	58.4%	27.00 (13.00-39.25)	0.176
Non-Saudi	47	41.6%	30.00 (18.00-59.00)	
Do you have children?				
Yes	79	69.9%	26.00 (15.00-39.00)	0.101
No	34	30.1%	34.00 (16.75-63.25)	
Number of children				
1-2	18	23.7%	27.50 (11.50-39.75)	0.487
3-5	47	61.8%	26.00 (15.00-36.00)	
More than 5	11	14.5%	26.00 (22.00-83.00)	
Educational level				
Did not receive any education	17	15.0%	39.00 (19.00-62.50)	0.688
Primary school	12	10.6%	25.50 (8.75-34.75)	
Middle school	11	9.7%	26.00 (9.00-47.00)	
High school	20	17.7%	29.50 (17.50-36.00)	
Diploma	8	7.1%	31.50 (24.50-61.50)	
Bachelors	36	31.9%	27.00 (15.25-44.75)	
Masters or PhD	9	8.0%	21.00 (10.50-41.00)	
Were you employed at the time of diagnosis?				
Yes	30	26.5%	27.50 (15.50-57.50)	0.891
No	83	73.5%	28.00 (15.00-42.00)	
Residence				
Owned	40	35.4%	29.00 (14.50-35.00)	0.614
Rental	61	54.0%	29.00 (17.00-49.50)	
I live with someone else	12	10.6%	21.00 (13.00-75.25	
Where do you live?				
Jeddah	91	80.5%	30.00 (14.00-47.00)	0.550
Outside Jeddah	22	19.5%	26.00 (17.75-34.00)	
How far is the hospital from your residence?				
Less 20 min	55	48.7%	31.00 (17.00-46.00)	0.343
20 min – 60 min	46	40.7%	25.00 (14.75-36.75)	
More than 60 min	12	10.6%	32.00 (14.25-86.50)	
How do you visit the hospital?				
I drive myself	13	11.5%	28.00 (21.50-66.00)	0.127
I have a personal driver	15	13.3%	19.00 (7.00-46.00)	
A relative or a friend drives me	65	57.5%	30.00 (15.50-37.50)	
I take public transportation	20	17.7%	30.50 (24.50-67.25)	
Household monthly income (Riyal)				
Less than 3,000	27	23.9%	28.00 (13.00-40.00)	0.375
3,000-5,000	26	23.0%	25.50 (16.50-89.00)	
5,001-10,000	24	21.2%	27.00 (20.25-59.50)	
10,001-15,000	16	14.2%	16.50 (9.25-33.50)	
15,001-20,000	8	7.1%	31.50 (25.50-45.00)	
More than 20,000	12	10.6%	34.00 (15.50-60.00)	
Do you have a family history of cancer?				
Yes	46	40.7%	30.50 (18.75-48.25)	0.274
No	67	59.3%	28.00 (13.00-39.00)	
Do you have insurance?				
Yes	21	18.6%	22.00 (10.00-37.50)	0.234
No	92	81.4%	29.50 (17.00-46.00)	
Comorbidities				
Yes	36	31.9%	33.00 (18.50-52.00)	0.258
No	77	68.1%	26.00 (14,00-39.50)	

**Figure 1:**
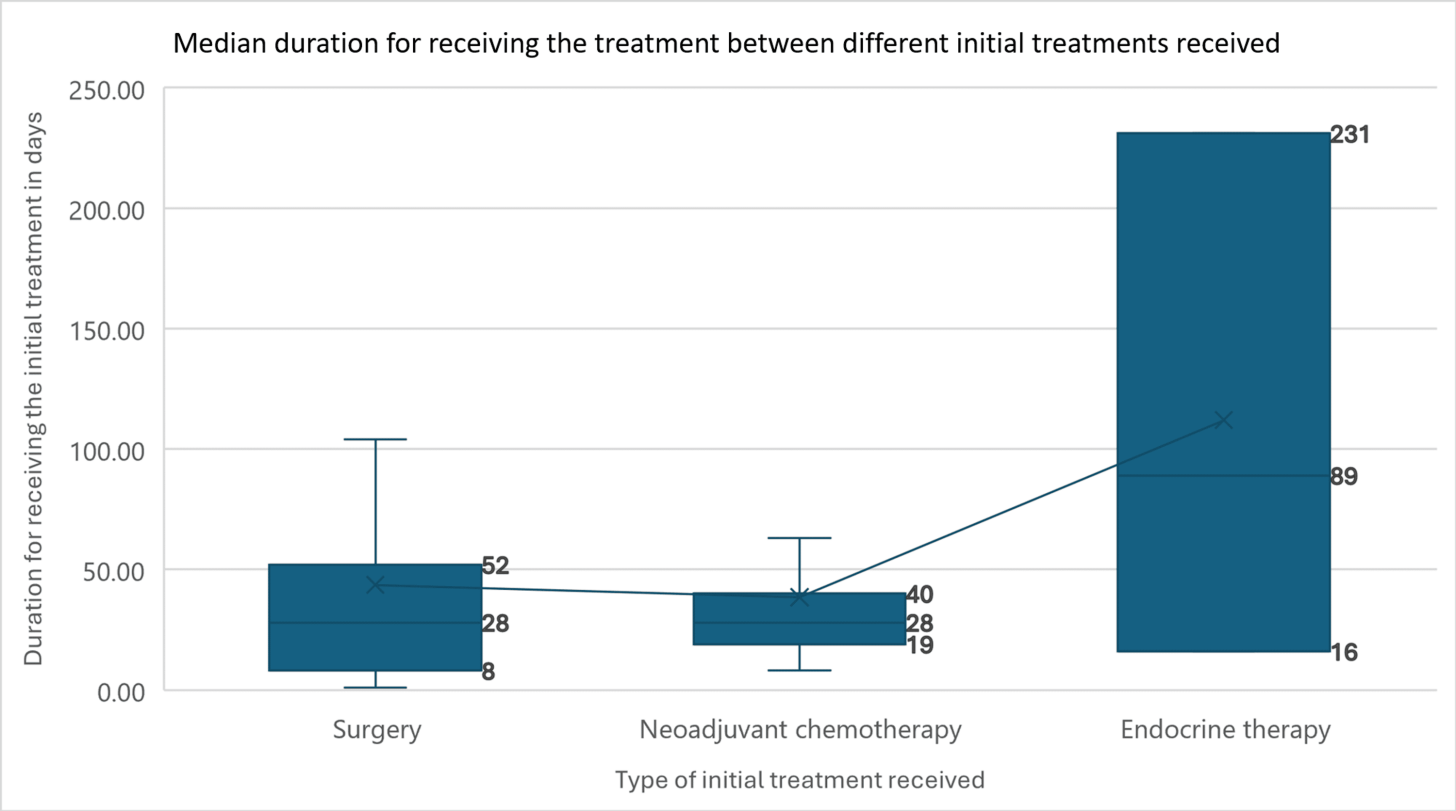
Median duration (in days) for receiving different breast cancer treatments The numbers in the figure represent the medians and interquartile ranges for the duration between diagnosis and treatment initiation in days between the different types of treatments received by our patients.

When assessing the relationship between the type of initial treatment received and the duration from diagnosis until the day it was received, patients who underwent surgery and those who took neoadjuvant chemotherapy had relatively similar durations. However, patients who had endocrine therapy had a longer duration compared to the rest (28.00 (8.00-52.00) vs. 28.00 (19.00-40.00) vs. 89.00 (16.00-231.00), p = 0.391). Figure 1 illustrates the mean duration for receiving each BC treatment.

## Discussion

This is the first study to investigate the causes of delays in diagnosis to treatment time among BC patients in Saudi Arabia. It is widely acknowledged in the literature that early treatment significantly reduces mortality rates. Research indicates that for every 60 days therapy is postponed, the mortality rate increases by 26% [18].

This current study found that the median duration of receiving the initial treatment was 28 days. Results of different literature vary; a study by Jaiswal et al. showed that the median time from diagnosis to treatment was 37 days [19]. Another study by McLaughlin et al. found the median to be 22 days [20]. A third study by Eriksson et al. found nearly similar results as the current study, with a median of 27 days [21]. This difference in the reported data by the literature further indicates that certain demographic and social factors could highly induce a delay in treatment initiation, as Jassem et al. demonstrated in their study that factors such as higher educational level and employment could decrease the period of delay to get the treatment [22]. This was also the case in our study, as patients without education demonstrated the longest durations for receiving the treatment.

Although some factors may have contributed to the duration between diagnosis and treatment, none showed any statistical significance throughout our results. Our study revealed that factors such as nationality, marital status, health insurance, and type of initial treatment may have contributed to delays in treatment decision-making. Notably, household income did not significantly impact treatment decisions in our context, given the comprehensive coverage provided by the Saudi Arabian healthcare system, as it is free. However, this contrasts with multiple approved articles, illustrating the remarkable bond between the patients’ monthly incomes and diagnosis-to-treatment time. A recent study has found that patients treated at private hospitals demonstrated a significantly lower delay period than those treated at public hospitals, emphasizing the importance of medical insurance [23]. Moreover, a retrospective study showed that women who had lower incomes and no insurance had higher rates of being diagnosed with later-stage disease [24, 25]. This indicates that patients are not only getting longer delays in receiving their treatment but receiving the diagnosis at a late stage, leading to more undesirable outcomes.

The type of initial treatment significantly influenced the delay in treatment initiation. Specifically, patients who received endocrine therapy first experienced longer delays compared to those undergoing surgery or neoadjuvant chemotherapy as the first treatment. This delay in the endocrine therapy group might be attributed to the time required for additional diagnostic testing, such as the Oncotype DX test, which evaluates the risk of cancer recurrence and helps guide treatment decisions. Waiting for test results can extend the time before starting endocrine therapy [26]. Moreover, patient preferences and clinical considerations could contribute to these delays. Some patients might refuse or be hesitant to undergo chemotherapy due to concerns about side effects, opting for endocrine therapy perceived as less aggressive. Additionally, patients selected for endocrine therapy might be deemed poor candidates for aggressive treatments due to underlying health conditions or advanced age, necessitating a more conservative approach and longer evaluation periods to ensure the chosen therapy aligns with their overall health status [27].

Distance is a crucial factor and could impact the time to receive treatment [28]. Our study demonstrated that patients outside the city had a more extended treatment duration than those inside. Also, patients who lived in the town but were away from the hospital had a longer period than those who lived nearby. Moreover, distance affects not only the timing of receiving treatment but also the choice of treatment. A study by Celaya et al. showed that patients who lived further from the hospital were less likely to undergo breast-conserving surgery [29]. This could be explained by the fact that patients who live outside the city weren't diagnosed early, or there needs to be more healthcare facilities outside the city. Additionally, patients who live far from the hospital get tired because of the distance and tend to go for a more aggressive management option to avoid travel.

Worldwide, there are racial differences that significantly affect treatment outcomes. A retrospective study conducted in South Carolina showed significant disparities in receiving treatment among different racial groups, with minority women experiencing delays in receiving either surgical treatment or advanced hormonal therapy [30]. Another study supports this, indicating that minority women at the same disease stage often do not receive prompt diagnosis and suggested therapy [31, 32]. However, we chose not to directly compare racial groups in receiving treatment for BC due to the complex and sensitive nature of racial disparities in healthcare. Instead, our study assessed the difference between Saudi and non-Saudi recipients in receiving treatment, demonstrating an insignificant relationship between them. This is because the Saudi government provides free financial coverage for treatment to all patients diagnosed in its hospitals.

Strengths and limitations

To our knowledge, this is the first study that investigated the influence of sociodemographic factors in delaying diagnosis to treatment time in BC patients in KSA. However, this study has some limitations. Firstly, some patients needed more data regarding the date of diagnosis, as they received the diagnosis from another institution and came to our tertiary center to start the proper treatment immediately. Additionally, the sample size needed to be bigger due to a high non-responder rate.

## Conclusions

Our study focused on demographic disparities in BC treatment initiation within the free healthcare system in KSA. While our findings showed that some variables were associated with a delay in treatment initiation, they did not show statistical significance. Nonetheless, factors including nationality and distance from the hospital suggest that there are more areas for investigation. Our study had its limitations, most importantly the relatively small sample size. This advocates for future research with a larger sample to be conducted in order to establish a better understanding of the different variables affecting the treatment initiation time. The implications of our findings extend beyond the academic realm, as such results could help improve accessibility and reduce treatment delays for BC patients, leading to better outcomes and higher rates of survival.

## Appendices

Appendix A

Research Questionnaire

**Table 4:** Research questionnaire

Questionnaire items
1- What was your marital status at the time of diagnosis?
Single
Married
Divorced
Widow
2- What is your nationality?
Saudi
Non-Saudi
3- What is your highest educational level?
Did not receive any education
Primary school
Middle school
High school
Diploma
Bachelors
Masters or PhD
4- Did you have any kids at the time of diagnosis?
Yes
No
5- If yes, how many kids did you have at the time of diagnosis?
1-2
3-5
More than 5
6- Did you smoke at the time of diagnosis?
Yes
No
7- Were you employed at the time of diagnosis?
Yes
No
8- If yes, has your employment status changed since you received the diagnosis?
No change
I retired
I resigned
My contract was terminated
I changed my position at work
9- If you were employed, did you need to take any extra time off work (other than your annual vacation)?
Yes
No
10- If yes, how many extra days did you take off?
Two weeks or less
More than two weeks
11- If you took any extra days off, did you receive sick leave pay for them?
Yes, it was paid
No, it was not paid
12- Who was the person who helped you the most during your treatment journey?
Husband
Parents
Children
Siblings
Relatives
Friends
I did not have any support
13- Do you own a home or rent or live with someone?
Owned
Rented
I live with someone else
14- Where is the place of your primary residence?
In Jeddah
Outside Jeddah
15- How long does it take for you to visit the hospital?
Less than 20 minutes
Between 20 to 60 minutes
More than 60 minutes
16- What mode of transportation do you usually use to visit the hospital?
I drive myself
I have a private driver
A friend or a relative drives me
I use public transportation
17- What is your monthly household income in Saudi riyals?
Less than 3000
3000-5000
5001-10000
10001-15000
15001-20000
More than 20000
18- Do you have medical insurance?
Yes
No
19- Did you need to take out a loan following your diagnosis?
Yes
No
20- Do you have any chronic illnesses or other comorbidities?
Yes
No
21- Do you have any history of cancer in your family?
Yes
No
